# Arthroscopic Palmaris Longus Tendon Bridging for Augmented Repair of Bursal‐Sided Partial Thickness Rotator Cuff Tears

**DOI:** 10.1002/atn2.70285

**Published:** 2026-10-09

**Authors:** Guo Zheng, ZunHan Liu, JunYan Yu, MoLong Chen, YunJiao Wang, ChenKe Zhang, Lin Ma

**Affiliations:** ^1^ Department of Sports Medicine First Affiliated Hospital of Army Medical University Chongqing China

## Abstract

The management of bursal‐sided partial‐thickness rotator cuff tears remains controversial. Both transtendon repair preserving the residual tendon and conversion to full‐thickness repair yield satisfactory outcomes. However, treating bursal‐sided partial‐thickness rotator cuff tears with significant tendon retraction because of delamination is challenging. Direct repair of the retracted bursal layer under high tension results in a high retear rate. The purpose of this technical note is to describe an arthroscopic method using the palmaris longus tendon for augmented repair of bursal‐sided partial‐thickness rotator cuff tears, aiming to promote biological healing and reduce the retear rate.

## Graphical abstract

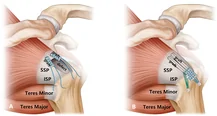

**VIDEO 1:** First, the glenohumeral joint cavity was accessed, and the articular surface of the rotator cuff was found to be intact and undamaged. Subsequently, the subacromial space was entered to perform subacromioplasty, decompression, and debridement. Partial injury of the bursal‐sided rotator cuff was observed, with a rotator cuff tissue defect formed by retraction after delamination. Despite thorough release, the bursal‐sided tear could not be manipulated to cover the rotator cuff footprint using tissue forceps. An arthroscopic ruler was used through the posterior and lateral portals to measure the anteroposterior and mediolateral dimensions of the defect, respectively. A suture hook loaded with the polydioxanone suture was used to perform mattress suturing at the myotendinous junction of the residual rotator cuff tissue. The woven sutures of the graft were guided through via the polydioxanone sutures, allowing the 2 blind ends of the graft to pass through the medial stump of the rotator cuff separately. A lateral‐row anchor was used to fix the graft to the lateral aspect of the greater tuberosity. A medial‐row anchor was implanted between the graft and the posterior rotator cuff stump, followed by side‐to‐side suturing between the graft and the posterior rotator cuff stump. After tying and tightening the sutures, the sutures were further secured to the lateral aspect of the greater tuberosity using additional lateral‐row anchors, and the surgery was completed. Video content can be viewed at https://doi.org/10.1002/atn2.70285.

Partial‐thickness rotator cuff tears (PTRCTs) are a common shoulder condition, presenting with pain, limited range of motion, and muscle weakness. Its prevalence in the population ranges from 13% to 40%.^1^ Arthroscopic repair is frequently recommended for Ellman grade 3 PTRCTs.^2^ Although the outcomes are comparable between transtendon repair that preserves the residual tissue and conversion to full‐thickness repair for bursal‐sided PTRCTs,^3^ certain patients continue to encounter persistent pain and functional limitations. Transtendon repair may create uneven tension across the cuff.^4^ When there is significant retraction of the bursal layer, performing a repair under high tension elevates the risk of retear. Conversion to full‐thickness repair is feasible for smaller tears. Nevertheless, in the case of larger bursal‐sided tears, conversion leads to a large or massive cuff defect that may not be suitable for direct repair, which compromises the outcomes.^5^

Managing bursal‐sided PTRCTs with substantial defects is challenging. Preoperative magnetic resonance imaging should assess features of delaminated tears, such as delamination type, retraction length of the bursal layer, intramuscular split length, anteroposterior tear length, and the degree of muscle fatty infiltration.^6^ Diagnostic arthroscopy is essential to confirm reparability. In some cases, even after adequate mobilization of the tendon and surrounding structures, the retracted bursal layer cannot be fixed anatomically without tension when the shoulder is abducted <60°.^7^ Even after footprint fixation, the poor quality and reduced volume of the residual tendon fibers contribute to a high retear rate. Superficial augmentation using the coracoacromial ligament has been reported but may not suffice for larger defects.^8^ The palmaris longus tendon, because of its accessibility, serves as an excellent graft source for rotator cuff reconstruction.^9^ This article details an arthroscopic technique for augmented repair of bursal‐sided PTRCTs using an autologous palmaris longus tendon graft. This technique aims to reduce repair tension, facilitate biological healing, and lower the retear rate by augmenting the repair with a palmaris longus tendon graft. A schematic of graft placement is shown in Figure 1. A comprehensive analysis of the advantages and limitations of our technique is presented in Table 1, with key technical considerations detailed in Table 2.

**FIGURE 1:**
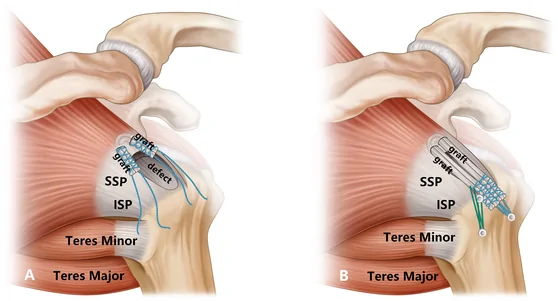
A schematic of graft placement. (A) Partial articular‐sided rotator cuff tear with retraction and delamination, resulting in a tendon tissue defect, whereas the underlying bursal‐sided rotator cuff tissue remains intact. The woven end of the graft is passed through the musculotendinous junction medial to the defect (not yet fixed). (B) After the graft is anchored and fixed at the lateral row, it covers and fills the defect. The medial‐ and lateral‐row anchors are used to perform side‐to‐side suturing between the graft and the posterior stump of the rotator cuff. (ISP, infraspinatus; SSP, supraspinatus.)

**TABLE 1:** Advantages and Disadvantages of Arthroscopic Procedures

**Advantages**	**Disadvantages**
• The palmaris longus autograft is superficial and easy to harvest from the ipsilateral limb with minimal morbidity	• Suitable primarily for medium‐to‐large partial‐thickness tears; may require combined techniques for massive tears
• The procedure is relatively straightforward and reproducible and has good clinical applicability	• Potential donor site complications
• The palmaris longus tendon typically has sufficient length and an adequate diameter for passage through cuff tissue	• The palmaris longus tendon is absent in approximately 20% of the population
• Provides a novel and effective bridging and augmentation strategy for delaminated tears	• Requires sufficient cuff tissue remnant for suture passage

**TABLE 2:** Pearls and Pitfalls

**Pearls**	**Pitfalls**
• For graft preparation, using the Krackow stitch technique commonly employed in ACL reconstruction is recommended	• Do not mistake the median nerve for the palmaris longus tendon: the nerve is whiter, softer, and typically round with visible epineural vessels; the tendon is yellower, tougher, typically oval, or flat without similar vessels
• Passing the graft through the rotator cuff at the musculotendinous junction provides the strongest fixation	• The tendon thickness is typically around 3 mm; it should be folded to achieve an adequate diameter
• If using a suture lasso to pass sutures through the cuff, passing the graft itself can be difficult; consider using a switching stick to dilate the passage or a BirdBeak suture passer	• Ethibond suture used for graft traction may break; therefore, using a high‐strength suture is recommended

ACL, anterior cruciate ligament.

## SURGICAL TECHNIQUE

### Patient Positioning and Preoperative Preparation

The surgical technique is shown in Video 1. The procedure is performed under general anesthesia with the patient in the lateral decubitus position. The affected limb is positioned in an arm limb positioner (AssistArm Limb Positioner, CONMED, Utica, NY) with 30° to 45° of abduction (Figure 2). Four standard arthroscopic portals are established (posterior, anterior, lateral, and anterolateral).

**FIGURE 2:**
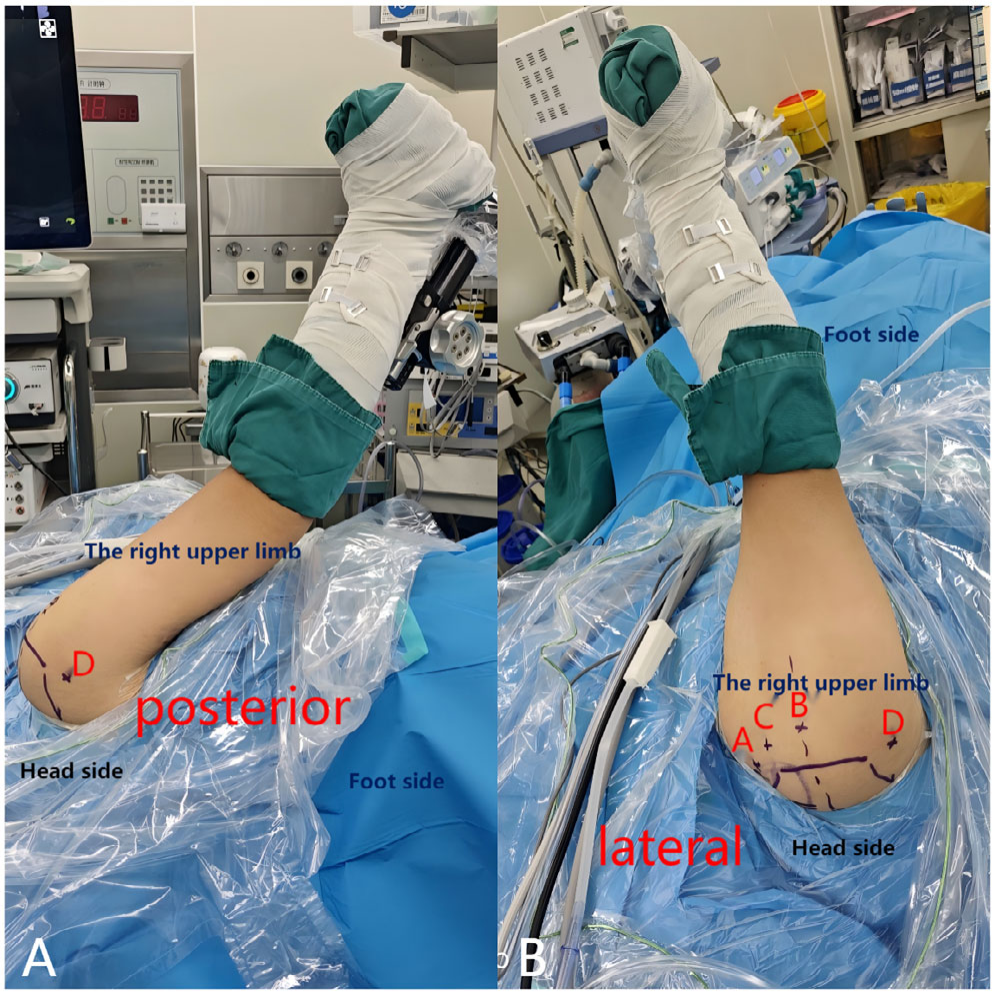
Patient positioning and portal placement. (A) Posterior view of the right shoulder. (B) Lateral view of the right shoulder. (A, anterior portal; B, lateral portal; C, anterolateral portal; D, posterior portal.)

### Portal Establishment and Diagnostic Arthroscopy

To begin, a 30° arthroscope (Smith & Nephew, Andover, MA) from the standard posterior and anterior portals is used for routine diagnostic arthroscopy of the glenohumeral joint to evaluate the long head of the biceps tendon, subscapularis, and other intra‐articular pathologies. If a subscapularis tear is present, it is repaired first. Capsular release and/or biceps tenotomy/tenodesis are performed as needed. The arthroscope is then withdrawn and redirected into the subacromial space via the posterior portal. An anterolateral portal is established. A thorough bursectomy and subacromial decompression are performed as indicated. The rotator cuff tear is assessed (Figure 3), and extensive releases are carried out if necessary to improve reparability.

**FIGURE 3:**
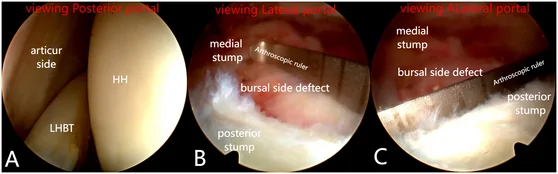
Thirty‐degree arthroscopic view showing intraoperative exploration and assessment of tear dimensions. (A) The articular side of the rotator cuff appears intact within the joint. (B) Arthroscopic measuring probe assessing the medial‐lateral retraction of the bursal‐sided tear. (C) Arthroscopic measuring probe assessing the anteroposterior length of the retracted bursal‐sided tear. (HH, humeral head; LHBT, long head of biceps tendon.)

### Palmaris Longus Tendon Harvest and Graft Preparation

The ipsilateral palmaris longus tendon is harvested. The tendon is palpated at the midpalmar wrist crease. A 1‐cm transverse incision is made over the palmar wrist crease. After blunt subcutaneous dissection, a high‐strength suture is woven through the distal tendon, which is then transected. The distal end is threaded into a tendon stripper, which is advanced proximally until it detaches at the musculotendinous junction. The harvested tendon is folded to create a 9‐cm‐long graft. The ends are similarly woven with high‐strength sutures, similar to the preparation of hamstring tendons for anterior cruciate ligament reconstruction (Figure 4).

**FIGURE 4:**
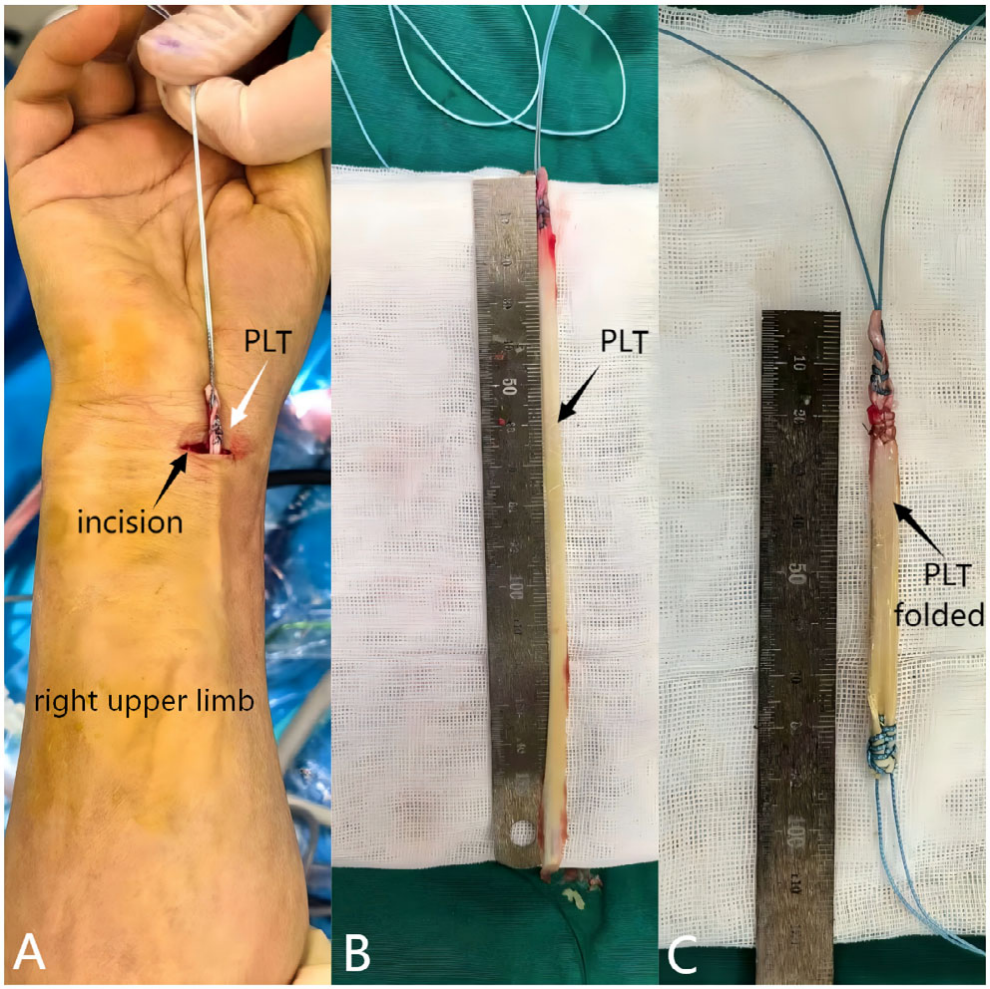
Palmaris longus harvest and suture preparation. (A) A 1‐cm incision over the palmar wrist crease; the distal tendon is woven and harvested using a tendon stripper. (B) Gross view of the harvested palmaris longus tendon. (C) The harvested tendon is folded to create a 9‐cm graft, and the ends are woven with sutures. (PLT, palmaris longus tendon.)

### Rotator Cuff Repair and Graft Implantation

Under visualization from the lateral portal, a suture passer is introduced via the posterior portal. A polydioxanone II guide suture is passed from outside to inside through the posterior aspect of the retracted bursal cuff layer at the musculotendinous junction. The intra‐articular end of the suture is retrieved through the anterolateral portal via a cannula. Another suture passer is introduced via the anterior portal, and a polydioxanone suture is passed from outside to inside through the anterior aspect of the retracted bursal cuff layer at the musculotendinous junction. Its intra‐articular end is also retrieved through the anterolateral portal. The sutures from the graft ends are sequentially shuttled through the cuff using the previously placed polydioxanone sutures via the anterolateral portal, completing a mattress suture configuration. The graft traction sutures are then pulled out through the anterolateral portal, pulling the graft limbs beneath the retracted cuff to the lateral aspect of the greater tuberosity. The footprint area and the intended graft path over the greater tuberosity are freshened with a burr. After adjusting the tension, the graft limbs are fixed to the lateral greater tuberosity using a knotless suture anchor (Smith & Nephew, Andover, MA). A 4.5‐mm medial‐row suture anchor (StarSportMed, Beijing, China) is placed at the medial edge of the graft path on the greater tuberosity to reinforce the graft fixation. Another 4.5‐mm medial‐row suture anchor (StarSportMed, Beijing, China) is placed between the graft and the residual cuff tissue to suture the posterior residual cuff tissue to the graft. Finally, the residual rotator cuff is fixed using a double‐row technique (Figure 5).

**FIGURE 5:**
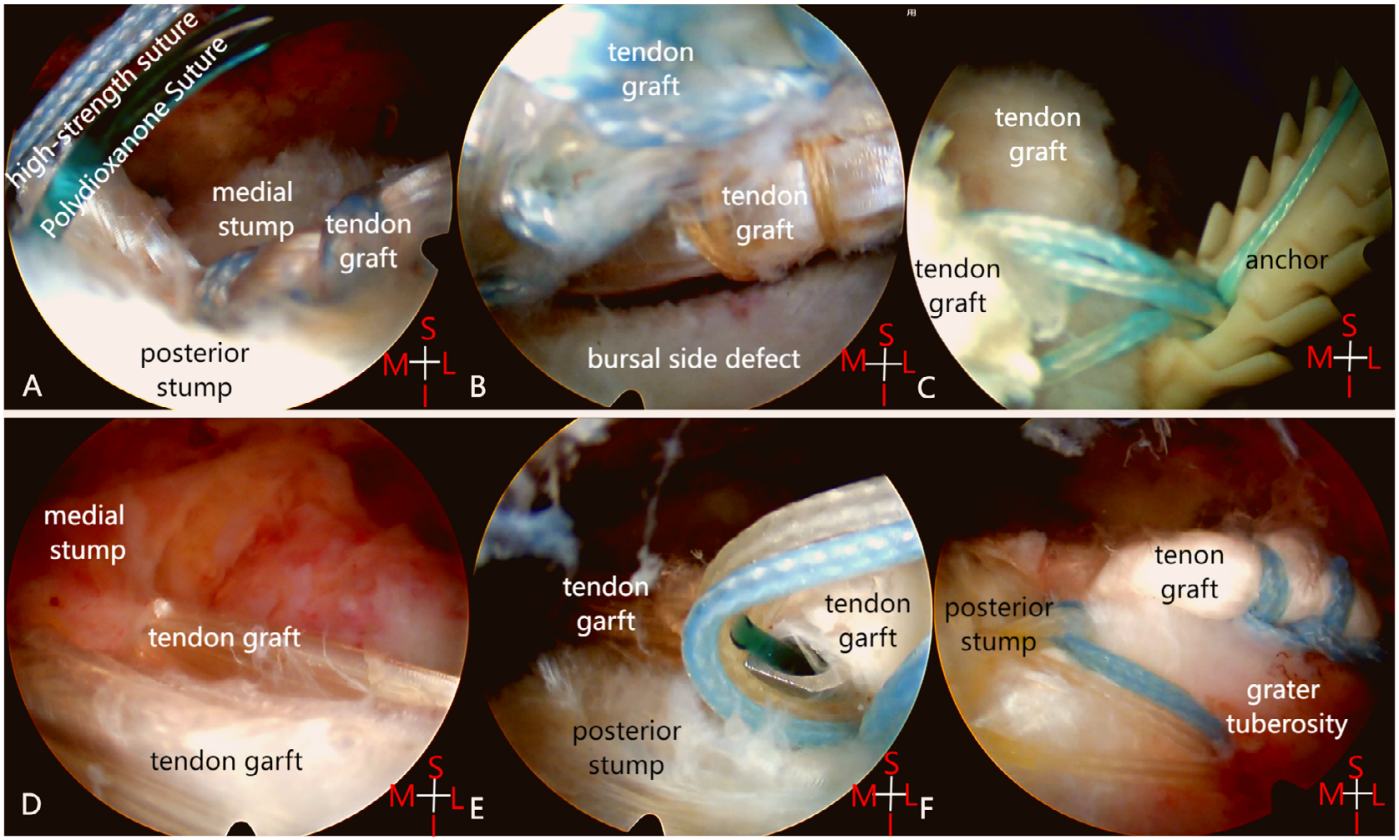
Steps of the arthroscopic procedure. (A) The graft is shuttled beneath the retracted cuff remnant using PDS sutures. (B) Both limbs of the graft have been passed beneath the cuff remnant, with the underlying defect from the retracted bursal‐sided tear visible. (C) Fixation of the graft (after suturing to the cuff remnant) to the lateral greater tuberosity using a knotless anchor. (D) Final view showing the tendon graft completely covering the defect. (E) The graft is sutured to the posterior cuff tissue using sutures from a medial‐row anchor passed with a PDS loop. (F) Final gross appearance after double‐row fixation of the residual cuff. Thirty‐degree arthroscopic view demonstrating the relevant structures. (I, inferior; L, lateral; M, medial; PDS, polydioxanone suture; S, superior.)

### Postoperative Rehabilitation Protocol

A shoulder abduction brace is strictly worn for the first 6 weeks postoperatively and removed only briefly for hygiene while supporting the hand. Patients perform daily grip strength exercises using a hand dynamometer and maintain an active range of motion for the wrist and elbow. Active‐assisted and active shoulder range‐of‐motion exercises commence at 6 weeks. Resistance training for the shoulder begins at 3 months. Preoperative and immediate postoperative magnetic resonance imaging scans are shown in Figure 6.

**FIGURE 6:**
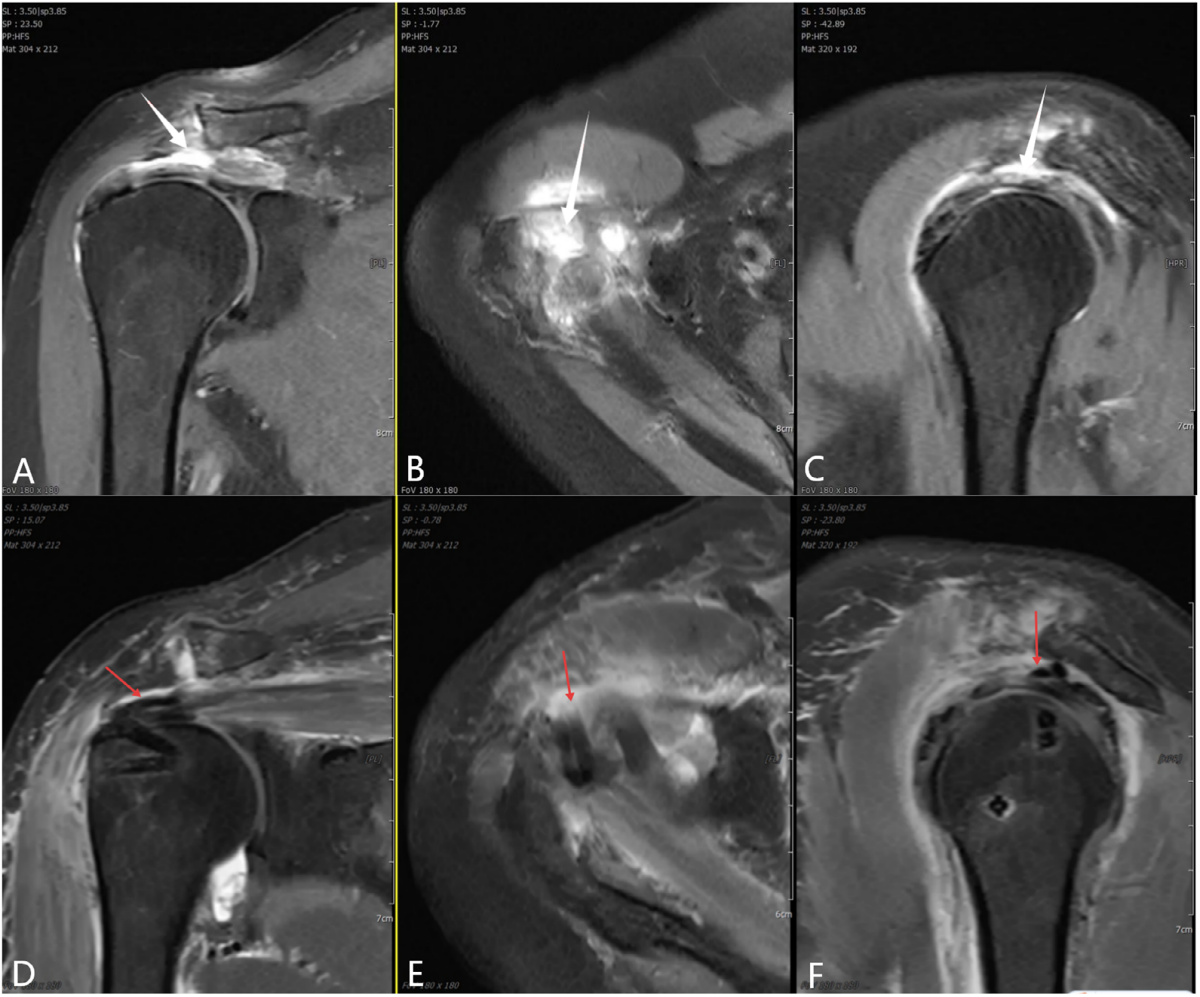
Preoperative and immediate postoperative MRI images. (A) Preoperative coronal view showing the defect from the retracted bursal‐sided partial delaminated tear (white arrow). (B) Preoperative axial view showing the defect (white arrow). (C) Preoperative sagittal view showing the defect (white arrow). (D) Postoperative coronal view showing the defect covered by the graft (red arrow). (E) Postoperative axial view showing the defect covered by the graft (red arrow). (F) Postoperative sagittal view showing the defect covered by the graft (red arrow). (MRI, magnetic resonance imaging.)

### Ethical Approval

Our clinical research was approved by the ethics committee of the First Affiliated Hospital of Army Medical University, (B2)2026KY005.

## DISCUSSION

This article describes an arthroscopic technique for augmented repair of bursal‐sided PTRCTs using an autologous ipsilateral palmaris longus tendon graft. This technique is indicated for patients with bursal‐sided PTRCTs exhibiting substantial defects, where direct repair is unlikely to achieve optimal cuff tension. It aims to reduce repair tension, promote biological healing, and lower the retear rate through palmaris longus tendon augmentation.

Historically, cadaveric studies suggested that intratendinous tears were the most common type of PTRCT.^10^ However, magnetic resonance imaging‐based clinical studies have challenged this: Kong et al.,^11^ reviewing imaging of 81 PTRCT patients, found bursal‐sided tears to be most prevalent at 72%, suggesting that their prevalence and clinical impact are underestimated, with a tendency to progress to full‐thickness tears. Kwon et al.^12^ defined delamination in PTRCTs as retraction of the articular or bursal layer with a recognizable horizontal gap between layers. In bursal‐sided delaminated tears, the separated intratendinous fibers exhibit varying degrees of retraction. Although the presence of delamination does not necessarily affect the overall outcomes of arthroscopic repair, the presence of medium‐to‐large, retracted delaminated tears may be a risk factor for poor outcomes after single‐row repair.^6^ Furthermore, large retracted defects from delaminated tears may not be amenable to in situ repair or conversion to full‐thickness repair. Given these issues, previous studies have employed patches for bursal‐sided PTRCTs, proposing the concept of augmentation using bioinductive collagen implants or autologous tendons rather than relying solely on the unhealthy residual tissue.^8^, ^13^, ^14^, ^15^ These studies offer surgeons a new patch augmentation strategy. Recently, the fascia lata, plantaris, peroneus longus, and biceps tendons have been used to augment repair of articular‐sided PTRCTs, yielding satisfactory functional outcomes for some patients.^16^, ^17^, ^18^ However, none matches the palmaris longus in terms of harvesting convenience, adaptability, and low donor site morbidity. Therefore, the autologous palmaris longus graft holds promise and research value for improving outcomes in chronic bursal‐sided PTRCTs or defects, especially compared with the more common techniques of conversion to full‐thickness repair and in situ repair.^19^ Regarding biocompatibility, the American Academy of Orthopaedic Surgeons released its latest guideline on rotator cuff injury management in August 2025. A significant change from the previous version is the “strong recommendation” for using bioinductive implants/scaffolds during repair to reduce retear rates and an explicit recommendation against using porcine xenografts.^20^ Compared with allografts, the palmaris longus autograft used in this technique may offer favorable biological integration because of its viable cellular components and nonimmunogenic nature,^21^, ^22^ which could theoretically promote healing while minimizing inflammatory reactions.

Currently, our short‐term clinical outcomes show promise for this technique in medium‐to‐large tears. However, further research and long‐term follow‐up are needed to evaluate its efficacy with expanded indications.

## DISCLOSURES

The authors (G.Z., Z.L., J.Y., M.C., Y.W., C.Z., L.M.) declare the following financial interests/personal relationships which may be considered potential competing interests: G.Z., Z.L., J.Y., M.C., Y.W., C.Z., and L.M. receives support from the Chongqing Innovative Medical Device Application Demonstration Project (CQEIC2024MDAD‐008), the National Natural Science Foundation of China (no. 82402876), and the Hundred Young Scholars Academic Cultivation Foundation of the First Affiliated Hospital of Army Medical University (no. 2025BQTJ‐6).

## References

[1] Oh JH , Lee YH , Lee TH , Jang SI , Kwon J . The natural history of high‐grade partial thickness rotator cuff tears: The conversion rate to full thickness tears and affecting factors. Clin Orthop Surg. 2020;12:514‐520. 10.4055/cios19167 PMC7683193 33274029

[2] Bi AS , O’Brien MJ , Waterman BR , Strauss EJ , Golant A . Management of partial‐thickness rotator cuff tears: Biologic and surgical interventions. Instr Course Lect. 2025;74:93‐116. 39745554

[3] Dalmas Y , Hao KA , Barret H , Mansat P , Bonnevialle N . Arthroscopic repair of bursal‐sided partial‐thickness rotator cuff tears: Literature review and meta‐analysis. Am J Sports Med. 2025;53:501‐507. 10.1177/03635465241239883 39790060

[4] Jeong JJ , Park SE , Ji JH , Lee HH , Jung SH , Choi BS . Trans‐tendon suture bridge rotator cuff repair with tenotomized pathologic biceps tendon augmentation in high‐grade PASTA lesions. Arch Orthop Trauma Surg. 2020;140:67‐76. 10.1007/s00402-019-03285-6 31616993

[5] Boileau P , Andreani O , Schramm M , Baba M , Barret H , Chelli M . The effect of tendon delamination on rotator cuff healing. Am J Sports Med. 2019;47:1074‐1081. 10.1177/0363546519835491 30943087

[6] Kim JH , Lee SK . Delaminated tears of the rotator cuff: MRI interpretation with clinical correlation. Diagnostics. 2023;13:1133. 10.3390/diagnostics13061133 PMC10047851 36980441

[7] Gerber C , Vinh TS , Hertel R , et al. Latissimus dorsi transfer for the treatment of massive tears of the rotator cuff. A preliminary report. Clin Orthop Relat Res. 1988;232:51‐61. 3383502

[8] Zhang Y , Zhou M , Hou J , et al. Arthroscopic autologous coracoacromial ligament augment technique for particle thickness of rotator cuff with at least 2 years follow‐up. Orthop Surg. 2023;15:2157‐2166. 10.1111/os.13697 PMC10432468 36905294

[9] Yammine K , Erić M . Morphometric analysis and surgical adequacy of palmaris longus as a tendon graft. A systematic review of cadaveric studies. Surg Radiol Anat. 2020;42:259‐267. 10.1007/s00276-019-02381-x 31741040

[10] Liu F , Dong J , Shen WJ , Kang Q , Zhou D , Xiong F . Detecting rotator cuff tears: A network meta‐analysis of 144 diagnostic studies. Orthop J Sports Med. 2020;8:2325967119900356. 10.1177/2325967119900356 PMC7003181 32076627

[11] Kong BY , Cho M , Lee HR , Choi YE , Kim SH . Structural evolution of nonoperatively treated high‐grade partial‐thickness tears of the supraspinatus tendon. Am J Sports Med. 2018;46:79‐86. 10.1177/0363546517729164 28949249

[12] Kwon J , Lee YH , Kim SH , Ko JH , Park BK , Oh JH . Delamination does not affect outcomes after arthroscopic rotator cuff repair as compared with nondelaminated rotator cuff tears: A study of 1043 consecutive cases. Am J Sports Med. 2019;47:674‐681. 10.1177/0363546518817764 30629459

[13] Bi AS , Morgan AM , O’Brien M , Waterman BR , Strauss EJ , Golant A . Partial‐thickness rotator cuff tears: Current concepts. JBJS Rev. 2024;12:1‐16. 10.2106/JBJS.RVW.24.00063 39186569

[14] Tokish JM , Shaha JS , Denard PJ , Mercuri JJ , Colbath G . Compressed biceps autograft augmentation of arthroscopic rotator cuff repair. Arthrosc Tech. 2022;11:e2113‐e2118. 10.1016/j.eats.2022.08.011 PMC9706130 36457381

[15] Colbath G , Murray A , Siatkowski S , et al. Autograft long head biceps tendon can be used as a scaffold for biologically augmenting rotator cuff repairs. Arthroscopy. 2022;38:38‐48. 10.1016/j.arthro.2021.05.064 PMC8665938 34126215

[16] Park SG , Seok HG . Use of an arthroscopic bridging graft for irreparable rotator cuff tears with the modified Mason‐Allen stitch using a tendon autograft. Arthrosc Tech. 2022;11:e857‐e861. 10.1016/j.eats.2021.12.044 PMC9134310 35646557

[17] Xu J , Li Y , Ye Z , et al. Biceps augmentation outperforms tear completion repair or in situ repair for bursal‐sided partial‐thickness rotator cuff tears in a rabbit model. Am J Sports Med. 2022;50:195‐207. 10.1177/03635465211053334 34779674

[18] Ma L , Liao YT , Wang ZY , Li HS , Tang KL , Zhou BH . Supraspinatus tendon reconstruction using fascia lata autograft for irreparable posterosuperior massive rotator cuff tears. Arthrosc Tech. 2023;12:e629‐e634. 10.1016/j.eats.2022.12.019 PMC10265272 37323798

[19] Strauss EJ , Salata MJ , Kercher J , et al. The arthroscopic management of partial‐thickness rotator cuff tears: A systematic review of the literature. Arthroscopy. 2011;27:568‐580. 10.1016/j.arthro.2010.09.019 21296545

[20] American academy of orthopaedic surgeons management of rotator cuff injuries evidence-based clinical practice guideline. Published August 18, 2025. aaos.org/rccpg2025. 10.7507/1002-1892.202511084 PMC12948511 41730726

[21] Weber SC . Editorial commentary: Mechanics versus biology: Both are relevant principles in understanding superior capsular reconstruction. Arthroscopy. 2021;37:1126‐1127. 10.1016/j.arthro.2020.12.227 33812517

[22] Li Y , Gou X , Liang Y , Ma L , Yuan C . Autologous tendon “Bamboo Raft” graft for reconstruction of massive irreparable rotator cuff tears. Arthrosc Tech. 2024;14:103266. 10.1016/j.eats.2024.103266 PMC11977160 40207321

